# The effect of dural release on extended laminoplasty for the treatment of multi-level cervical myelopathy

**DOI:** 10.1186/s12891-019-2554-8

**Published:** 2019-05-01

**Authors:** Yuwei Li, Xiaoyun Yan, Wei Cui, Yonghui Zhang, Cheng Li

**Affiliations:** Department of Orthopedics, Luohe Central Hospital of Orthopedics, No. 54, People’s Road, Luohe City, 462000 Henan Province China

**Keywords:** Dural release, Extended laminoplasty, Multi-level cervical myelopathy

## Abstract

**Objective:**

The effects of dural release on extended laminoplasty for the treatment of multi-level cervical myelopathy were explored and discussed.

**Method:**

Patients, who underwent extended laminoplasty combined with dural release for the treatment of multi-level cervical myelopathy (35 cases, group A), were compared with patients who underwent simple extended laminoplasty (38 cases, group B). The JOA score, improvement rate, VAS score, distance of retroposition of the spinal cord, cervical lordosis were compared between the two groups.

**Results:**

Dural laceration occurred to five patients during surgery, three in group A and two in group B; cerebrospinal fluid leakage occurred to five patients, three in group A and two in group B. All patients were followed up for 10 to 48 months (mean 20.3 months). JOA scores and VAS scores in the last follow up period were significantly improved in the two groups than preoperative scores (*p* < 0.05). The improvement rate and JOA scores in group A were significantly higher than group B, while VAS scores in group A were significantly lower than group B (*p* < 0.05). There were no significant differences in cervical lordosis in the two groups in the last follow up (*p* > 0.05), and the distance of retroposition of the spinal cord in group A was higher than B (p < 0.05). No shut-up of the ‘door’ of vertebral lamina occurred in the period of follow-up.

**Conclusion:**

Dural release on extended laminoplasty can achieve retroposition of the spinal cord for multi-level cervical myelopathy, which is more effective than simple extended laminoplasty.

## Introduction

Multi-level cervical myelopathy is a progressive disease that needs surgery for improvement, and it is a potential destructive nerve disorder resulting from spinal cord injury that is related to degeneration of the discs and other supporting spinal column structures [1]. In many patients, neurologic deterioration is the characteristic of the natural history of cervical myelopathy, therefore, surgery is frequently advocated by surgeons [2, 3].

There are various surgical procedures used in the treatment of patients with multi-level cervical myelopathy, including cervical laminoplasty, cervical laminectomy, cervical laminectomy and fusion, anterior cervical discectomy and fusion, corpectomy, etc. [4–7]. Posterior cervical laminoplasty is one of the most effective methods for the treatment of multi-level cervical myelopathy, but there were some drawbacks for some patients combined with ossification of the posterior longitudinal ligament, such as limitation of retroposition of the spinal cord and poor efficacy [8–10].

It is rare for the report about whether dural release will affect retroposition of the spinal cord and the efficacy of cervical myelopathy after posterior extended laminoplasty. Hence, we conducted a retrospective study to evaluate the effect of dural release on this surgery. We analyzed the data of 35 patients who underwent extended laminoplasty with dural release from September 2012 to December 2014, and compared with the data of 38 patients who underwent simple extended laminoplasty from April 2011 to April 2012.

## Materials and methods

### Patients

Thirty-five patients, who underwent extended laminoplasty with dural release for the treatment of multi-level cervical myelopathy, were divided into group A. Thirty-eight patients, who underwent simple extended laminoplasty, were divided into group B. There were no significant differences in sex, age, disease course, involved segments, complications, preoperative cervical lordosis, Japanese Orthopaedic Association score (JOA score), visual analog scale (VAS score) between the two groups (*p* > 0.05) (Tables 1 and 2).

**Table 1:** Comparison of therapeutic effect assessment index between the two groups before and after surgery (^−^x ± s)

Group	Cases	VAS score			JOA score			
		Preoperative	Last follow-up	t and *p* value	Preoperative	Last follow-up	t and p value	Improvement rate (%)
A	35	4.63 ± 1.88	0.81 ± 0.66	t = 22.158, p < 0.001	8.25 ± 1.36	15.59 ± 3.06	t = 43.066, p < 0.001	82.79 ± 11.57
B	38	4.78 ± 1.35	2.23 ± 0.97	t = 16.310, p < 0.001	8.29 ± 1.68	12.57 ± 3.01	t = 14.933, p < 0.001	47.35 ± 9.79
t and p value		t = 1.659, *p* = 0.107	t = 11.596, p < 0.001		t = 0.833, *p* = 0.423	t = 20.496, p < 0.001		t = 24.762, p < 0.001

**Table 2:** Comparison of imaging index between the two groups (^−^x ± s)

Group	Cases	Distance of retroposition of the spinal cord (mm)	Cervical lordosis		
			Preoperative	Last follow-up	t and p value
A	35	3.63 ± 2.06	19.55 ± 6.81	16.78 ± 7.92	t = 1.536, *p* = 0.142
B	38	2.05 ± 0.93	18.94 ± 8.73	18.22 ± 5.79	t = 1.784, *p* = 0.089
t and p value		t = 7.256, p < 0.001	t = 0.223, *p* = 0.854	t = 0.359, *p* = 0.782	

Group A: There were 19 males and 16 females, who were aged from 25 to 77 years old with an average age of 59.2. The disease course was 5–38 month with an average course of 13.8 month. Lesions of 18 cases involved C3/4, 33 cases involved C4/5, 35 cases involved C5/6, and 17 cases involved C6/7. Six patients (17.1%) combined with hypertension, and eight patients (22.9%) combined with diabetes.

Group B: There were 20 males and 18 females, who were aged from 24 to 78 years old with an average age of 61.3. The disease course was 6–37 month with an average course of 13.1 month. Lesions of 25 cases involved C3/4, 37 cases involved C4/5, 38 cases involved C5/6, and 17 cases involved C6/7. Seven patients (18.4%) combined with hypertension, and eight patients (21%) combined with diabetes.

### Inclusion and exclusion criteria

Inclusion criteria: 1. Patients accorded with diagnostic criteria of cervical myelopathy at the second National Symposium on Cervical Spondylopathy [11], who had progressive limb sensory, motor or sphincter dysfunction. 2. MRI and CT showed ossification of posterior longitudinal ligament in C3–7 and multi-segment compression of the spinal cord.

Exclusion Criteria: 1. Localized ossification of the posterior longitudinal ligament. 2. Disappearance of cervical lordosis or cervical kyphosis. 3. Cervical instability.

### Surgery

The surgeries of the two groups were performed by the same group of surgeons. Patients were taken prone position under general anesthesia, and the neck was flexed to avoid wrinkles of the rear of the skin so as to reduce the overlap of intervertebral disk and increase laminae interval space.

**Fig. 1:**
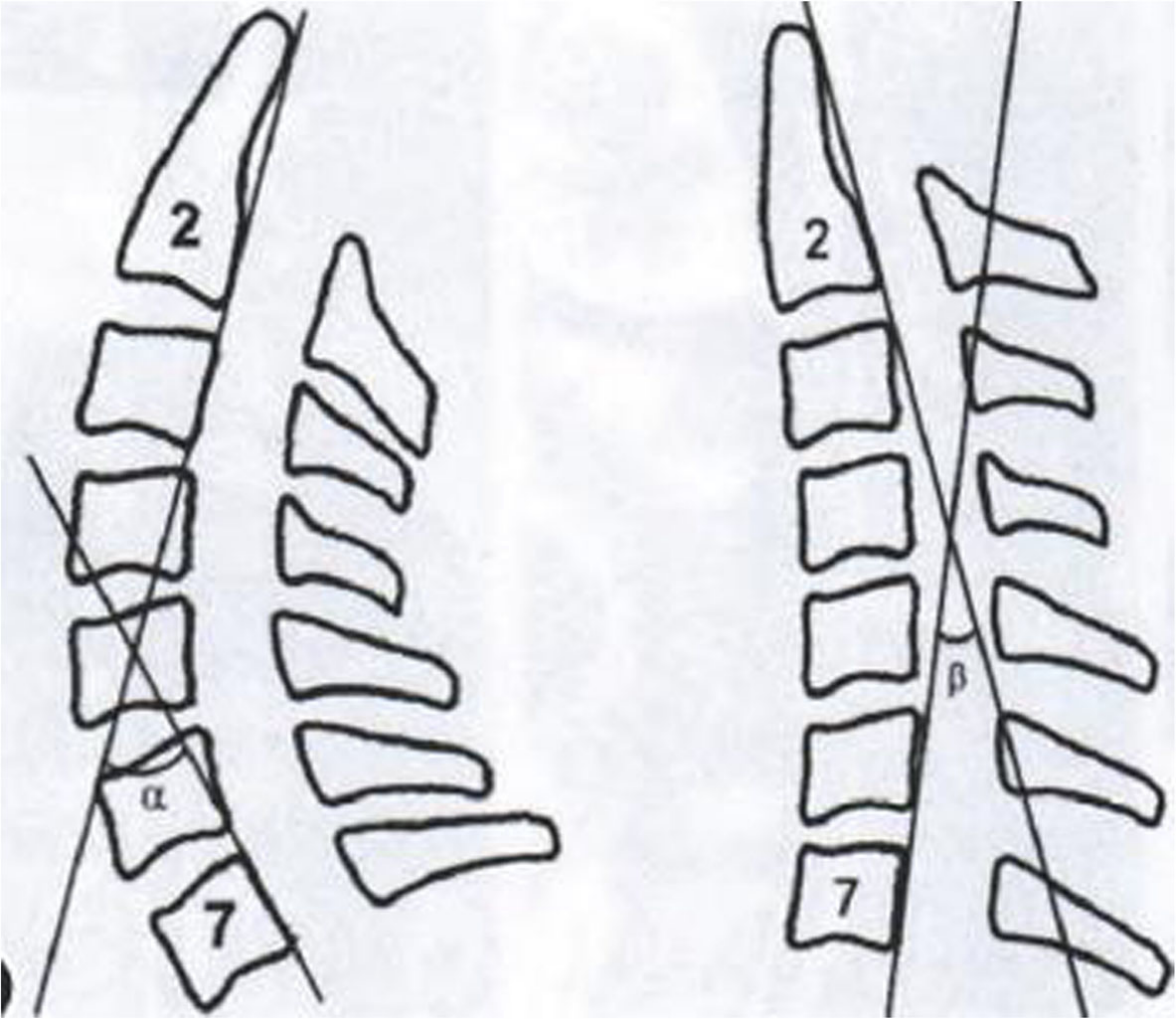
The diagram of spinal cord shift and expansion

For simple extended laminoplasty, C3–7 spinous process was shortened, a hole was made at the base of spinous process, and the side with heavier symptoms was used as the open side. Cortical bone of outer layer of the lamina was removed at the lateral border of vertebral lamina and lamina groove as door spindle. Vertebral lamina was removed and opened at the open side of lamina groove. The string was led through the spinous process, and sutured to the joint capsule of posterior lateral joint and attachment point of tendon. Lamina was lifted up to 60°, and the suture was knotted and fixed to soft tissue of articular process. The open segment of the nerve root canal was expanded 2-5 mm, so that the nerve root had certain flexibility [12–14] (The nerve root was touched by nerve stripping, and made a slight move). The diagram of spinal cord shift and expansion was shown in Fig. 1.

For extended laminoplasty with dural release, on the basis of single open-door, spinal dural of the decompression segment was elected to crash and the strand was released by nerve stripping. When the strand was tight, it was cut by meningeal scissors. C4–6 nerve root canal at the open side was expanded for 2–5 mm, then L-shaped hook of the nerve stripping was stick closely to ventral spinal dural and put into the nerve root canal carefully to explore the adhesion degree of anterior spinal dural and separate the adhesion. Attention should be paid to avoid excessive provoking spinal dural. Soft tissue adhesions such as strips and cords should be released. If there was dural calcification with osseous adhesion during surgery, the dorsal spinal dural calcification should be thoroughly decompressed. The ventral spinal dural calcification should not be treated to avoid injury and tearing of the spinal dural.

### Postoperative treatment

Neck collar was used for fixation for 8 week in the two groups. On the second day after surgery, the upper extremities were active/passive fisted, and made function trainings, such as hip, knee and ankle joint flexion at the lower limbs to promote the recovery of weight bearing and walking. One week later, patients could stand up and have out of bed activities.

### Efficacy evaluation

JOA scores were used to evaluate the nerve functions, and the improvement rate was calculated. VAS scores of neck and shoulder pain were used to evaluate the neck and shoulder pain improvement.

$$ \mathrm{Improvement}\ \mathrm{rate}=\frac{\mathrm{Follow}\hbox{-} \mathrm{up}\ \mathrm{JOA}\ \mathrm{score}\hbox{-} \mathrm{Preoperative}\ \mathrm{JOA}\ \mathrm{score}}{17\hbox{-} \mathrm{Preoperative}\ \mathrm{JOA}\ \mathrm{score}}\mathrm{x}100\% $$

Twelve months after surgery, MRI was conducted for measuring the distance of retroposition of the spinal cord. Midline sagittal T2-weighted images were selected, and Zoomagic software (Apps Rocket, England) was used to measure the distance between the middle point of vertebral posterior and the spinal posterior in each segment. The difference between pre-operation and post-operation was calculated, namely the distance of retroposition of the spinal cord of each segment. The mean value of all segments was the distance of retroposition of whole spine.

**Fig. 2:**
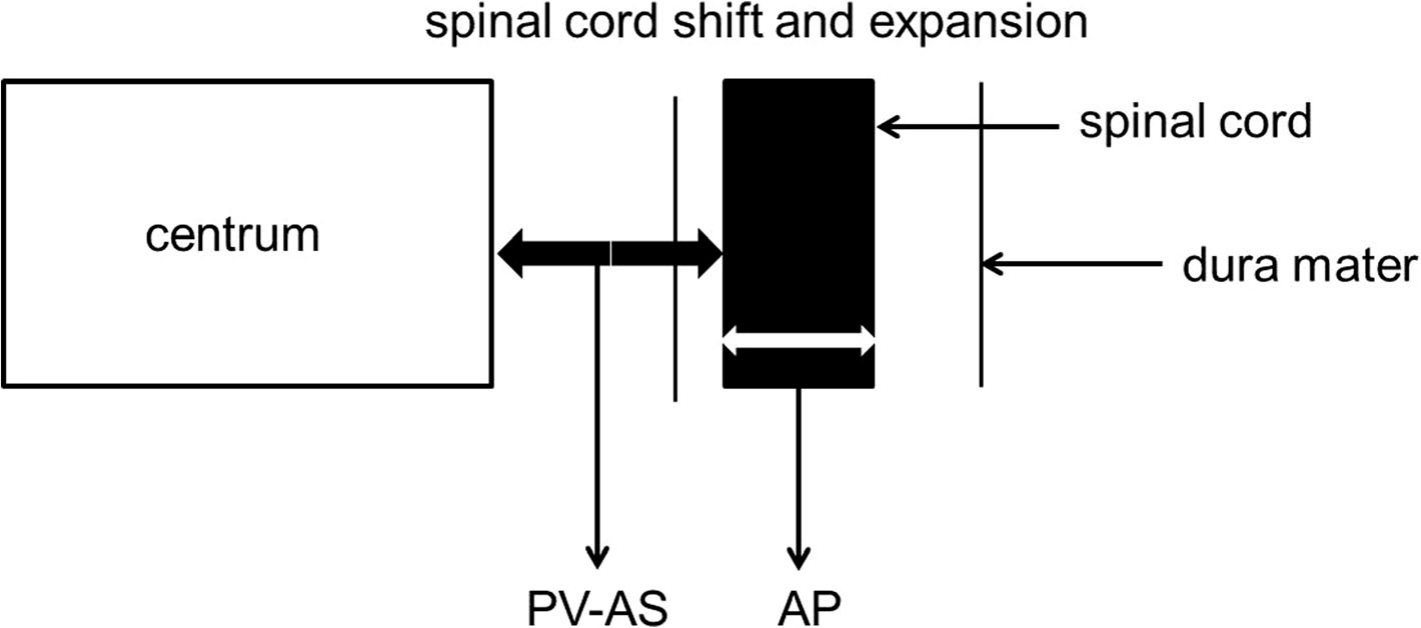
The angle between C2 and C7 was measured as “cervical lordosis”

X-ray was conducted for preoperative and postoperative cervical lateral position, and Zoomagic software was used to measure the angle of tangent of C2 and C7 posterior wall, namely the cervical lordosis (Fig. 2) [15].

CT was conducted for preoperative and postoperative cervical transverse position to measure whether there was ossification of posterior longitudinal ligament before surgery, the degree of the opening of vertebral lamina, and whether there was shut-up of the ‘door’ of vertebral lamina after surgery.

### Statistical analysis

We used SPSS 16.0 for the data analysis. Measurement data were expressed as mean ± standard deviation. Normality was tested using Kolmogorov-Smirnov test. Comparison between groups was analyzed by independent sample t-test, and comparison of preoperative and postoperative group was analyzed by paired sample t-test, test level ɑ = 0.05.

## Results

### Complications

All the patients successfully underwent the operation. Dural laceration occurred to five patients during surgery, three in group A and two in group B. In group A, the cleft located at the lateral anterior of spinal dural, and the spinal dural was unsutured. When closed the incision, all levels of organizations were tightly sutured. In group B, the cleft located at the dorsal side of spinal dural. 5–0 nylon suture was used for suture the spinal dural. Cerebrospinal fluid leakage occurred to five patients, three in group A and two in group B. They recovered after a series of treatments, such as local sandbag compression, reverse trendelenburg prone position, and replacement of electrolytes. Complications, such as incision infection and C5 nerve root palsy, didn’t occur to patients in the two groups after surgery.

### Follow-up

All patients were followed up for 10 to 48 months (mean 20.3 months). JOA scores and VAS scores in the last follow up period were significantly improved in the two groups than preoperative scores (*p* < 0.05). The improvement rate and JOA scores in group A were significantly higher than group B, while VAS scores in group A were significantly lower than group B (p < 0.05) (Table 1).

### Imaging diagnosis

**Fig. 3:**
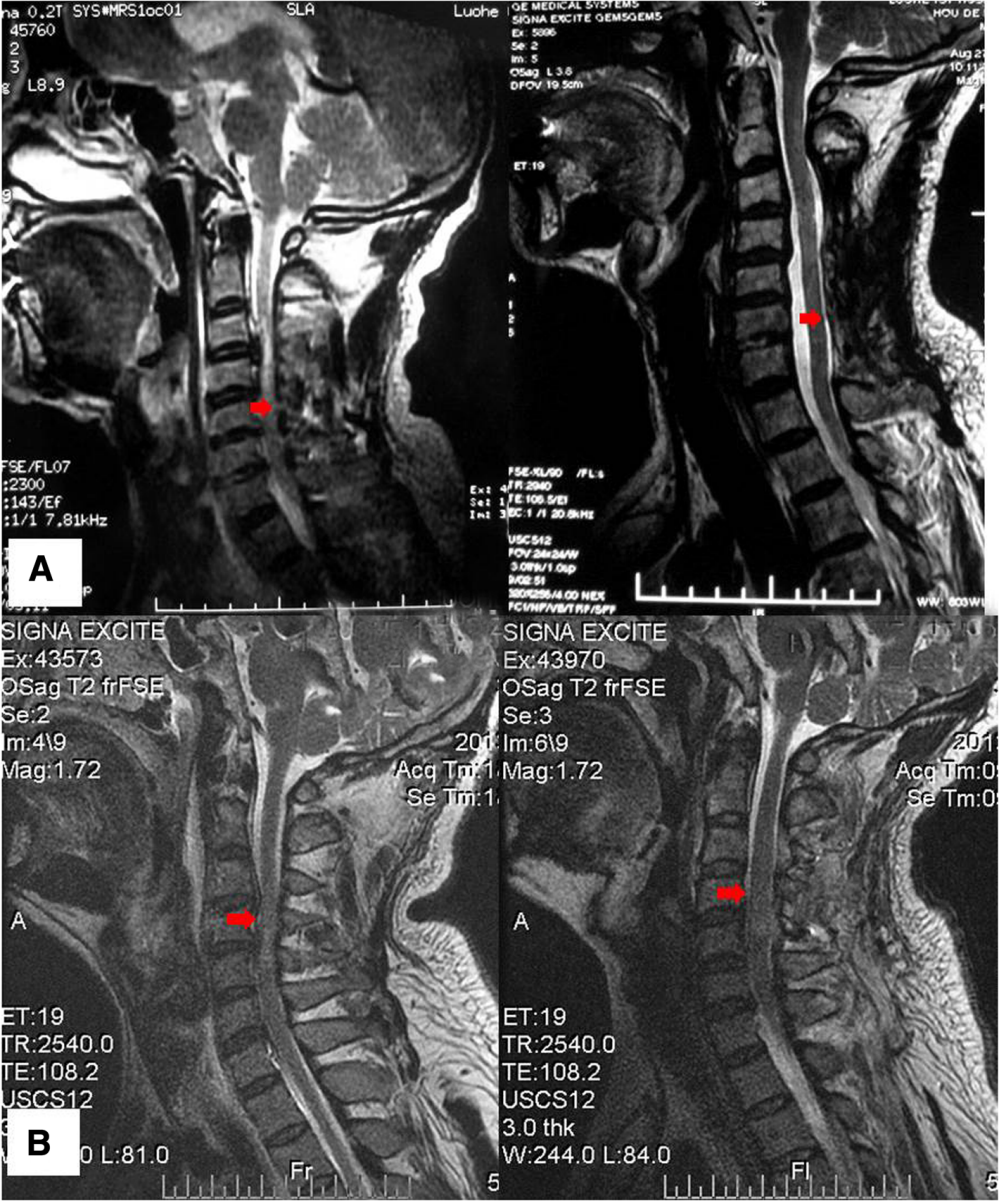
The shift of spinal cord in the group Aand group B. **a** The spinal cord of a 53-year-old man in group A who had C3–7 cervical myelopathy. **b** The spinal cord of a 41-year-old man in group B who had C3–7 cervical myelopathy

There were no significant differences in cervical lordosis in the two groups in the last follow up (*p* > 0.05), and the distance of retroposition of the spinal cord in group A was higher than B in the last follow up (t = 7.256, *p* < 0.001) (Table 2). No shut-up of the ‘door’ of vertebral lamina occurred in the period of follow-up (Fig. 3).

## Discussion

There are two main decompression principles of cervical myelopathy after posterior extended laminoplasty [15–19]. One is to directly remove the compression of spinal cord, and the other is to achieve retroposition of the spinal cord by using ‘bow string’ principle to avoid anterior compression, including anterior intervertebral disc, osteophyte, and hypertrophic and ossific posterior longitudinal ligament. But retroposition of the spinal cord is restricted by many factors, including cervical lordosis, whether nerve root canal expanded or not, and the degree of the opening of vertebral lamina [20–22]. It is rare for the report about whether dural release will affect retroposition of the spinal cord and the efficacy of cervical myelopathy after posterior extended laminoplasty. Hence, we conducted retrospective study to evaluate the effect of dural release on this surgery.

The resolution of MRI is high in soft tissues, and MRI is safe, non-invasive, and repeatable, which can directly observe the bony and non-bony structures in the spinal cord and spine. T2-weighted images can clearly show the margin and morphology of spinal cord and vertebrae, and the sagittal T2WI can clearly show cervical lordosis that can be a good way to measure the distance of retroposition of the spinal cord after cervical myelopathy after posterior extended laminoplasty. In this study, we chose to measure the distance of retroposition of the spinal cord by median sagittal T2WI. Radcliff et al. [23] thought the distance of retroposition of the spinal cord was related with nerve recovery. However, Tashjian et al. [24] found the distance of retroposition of the spinal cord had no relationship with the improvement rate after laminectomy of cervical myelopathy, but related with individual factors, such as age and the degree of cervical spondylosis. The reasons for the inconsistencies in these reports were, in our opinion, related with age, disease course, preoperative JOA score, MRI signal changes, the area of spinal cord compression, and operation methods and techniques [25], and retroposition of the spinal cord is only one of these reasons. The surgeries in patients of the two groups were all performed by the same group of physicians, and only group A underwent dural release. Results showed the distance of retroposition of the spinal cord, JOA score, improvement rate, and VAS score in group A were significantly better than group B.

Dural release on extended laminoplasty has many advantages. When expanded the sagittal diameter of cervical spinal canal, the restraint stress of adhesive tissue (strands posterior to spinal dural) to spinal dural was removed, which was beneficial for the retroposition of spinal dural and spinal cord. Besides, it could release part of the adhesive tissue anterior to spinal cord, which was beneficial for the retroposition of spinal cord. When lateral anterior of spinal dural was visible on the open side, C4/6 nerve root canal was expanded for the retroposition of spinal cord [26].

However, there were some disadvantages of this technique. It could only release soft tissue adhesion, such as strands and scar, and had risks of dural laceration when release bone adhesion with dural calcification. Bone adhesion anterior to spinal dural could not be released. Because it needed to expand C4/6 nerve root that would increase the risk of intravertebral venous plexus hemorrhage and prolonged the operation time. Meanwhile, if there was bone adhesion with dural calcification, dorsal side of dural calcification should be completely decompressed and the decompression area should be larger than calcification area. No treatment was conducted for ventral calcification to aviod injury or dural laceration. Anterior cervical decompression and calcified floating were depended on postoperative function recovery of spinal cord.

## Conclusion

For multi-level cervical myelopathy, sufficient dural release on extended laminoplasty was beneficial for retroposition of the spinal cord and could improve the curative effect. This study was a retrospective study and the sample size was small. Long-term follow-up of large samples, and multivariate analysis were needed to further clarify the effect of dural release on the efficacy.

## Abbreviations

JOA score: Japanese Orthopaedic Association score
VAS score: visual analog scale
